# Bluetooth Load-Cell-Based Support-Monitoring System for Safety Management at a Construction Site

**DOI:** 10.3390/s22103955

**Published:** 2022-05-23

**Authors:** Haksun Kim, Sehwan Park, Minkyo Yeom, Hakbo Shim, Soonjeon Park, Junkyeong Kim

**Affiliations:** 1 Safety Inspection for Infrastructure Laboratory (SIIL), Advanced Institute of Convergence Technology (AICT), 145 Gwanggyo-ro, Yeongtong-gu, Suwon-si 16229, Gyeonggi-do, Korea; skykhsui90@snu.ac.kr (H.K.); sehwan0721@snu.ac.kr (S.P.); tomsmith850918@snu.ac.kr (M.Y.); 2 Research & Development Institute, LOTTE Engineering & Construction, 38, Naruteo-ro 10-gil, Seocho-gu, Seoul 06515, Korea; hakbo.shim@lotte.net (H.S.); soonjeonpark@lotte.net (S.P.)

**Keywords:** system support, Bluetooth load-cell, remote load-monitoring, collapse

## Abstract

At construction sites, temporary facilities have caused continuous collapse accidents, causing damage to human life. If the concrete placing height is high and the worker is pushed into one place at the time of placing, the working load may be exceeded and a collapse accident may occur. In order to solve this problem, in this research, we developed a monitoring load-measurement program based on a Bluetooth wireless load cell (load-cell sensor) so that the load can be converted to digital and the numerical value can be confirmed by the pressure sensor. The load cell using Bluetooth was designed and manufactured according to the support. Then, the performance was verified through 3D finite element analysis by modeling and experimental tests. In addition, we constructed a system to generate notifications and warnings step by step when the load is close to a dangerous load, confirmed the load distribution pattern by position, and established a method to confirm real-time data numerically and graphically. Finally, we evaluated the practical application of the load-monitoring system using field-test data using a wireless load-cell.

## 1. Introduction

When injecting concrete, only temporary structures need to support the concrete’s own weight and impact load. However, due to deterioration of temporary materials and improper installation, the structure may become unstable, and the concrete casting site may collapse. In addition, even if the safety factor of various temporary structures is taken into consideration at the time of design and the safety factor is reviewed, it is not possible to completely control the load change due to various variables during concrete placement. Sensor-based measurement and monitoring systems are needed at construction sites to detect formwork and structural deformation and prepare for collapse. In the existing construction method, a method of assigning a site manager to perform visual measurement at the time of concrete placement is adopted, but the view is that it is insufficient as a risk countermeasure method for the risk of collapse. Therefore, in this research, we developed wireless sensor load-monitoring technology that can quickly grasp the entire process and construction status and applied it to the site to improve the safety of construction.

Large and small collapse accidents have occurred at construction sites, and casualties are increasing. According to construction accident statistics, accident rates in all industries are declining continuously, but accident rates in the construction industry are on the rise [1].

Referring to Table 1, the number of disasters in the construction industry—which peaked in 2012—seemed to be decreasing, but it showed a trend of increasing year by year [1].

**Figure 1:**
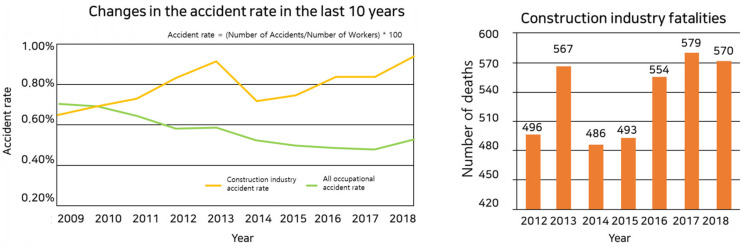
Accident rate and number of deaths in construction [2].

In addition, according to Korea’s construction accident statistics, the accident rate of all industries continues to decrease, as shown in Figure 1, while the accident rate of the construction industry is on the rise. Despite efforts to reduce these accidents, many deaths occur every year [2].

In particular, according to the working-level guidelines published by the Ministry of Employment and Labor, 22.8% (488 out of 2134) died from serious accidents based on scaffolding work over the past five years.

The most common cause of these accidents is the lack of or improper installation of temporary fixtures and system supports. In particular, the result of system-support installation is often different from the original plan due to the lack of manpower of safety managers and cost reduction of construction materials. In addition, compared to permanent facilities, it is judged that the investment and interest in on-site structural safety review, material quality inspection, installation supervision, and worker safety equipment are insufficient [3,4,5,6].

However, the most dangerous thing is that sudden collapse accidents cannot be prevented because there is no intuitive way to know which loads are at risk of collapse.

Therefore, in the initial design and planning stage, all potential dangers should be identified, and preventive measures should be taken. However, the safety planning used in the construction industry has certain shortcomings. Construction safety plans are often carried out separately from the initial planning [7]. Therefore, in many construction projects, safety experts do not actively participate in the creation or revision of the construction plans, and their roles are limited to the inspection of construction sites. The safety analysis of a construction site depends greatly on the manual work of individual safety managers or supervisors in identifying potential safety risks. However, manual safety inspections are generally labor intensive, and thus are prone to errors owing to the complex and changing characteristics of construction projects. Moreover, designers have paid limited attention to safety because they often fail to understand how safety is affected by on-site work [8].

Today, electrical-load transducers are widely used in various fields. The development of this type of transducer began about 40 years ago, when it was limited to load control of 10 tons or more and began to be used as a partially improved mechanical device. After decades of research and problem solving, it was developed until the use of precision transducers with an accuracy of less than 0.02% of maximum load became common.

Previous studies have demonstrated higher safety management through the use of a performance certification system and proper management of the construction materials, as well as construction-site monitoring through the installation of an inclinometer used to control the safety of the support pillars using information technology [9,10,11,12,13]. Other studies have conducted remote measurements through the development of wireless custom load-cells [14,15].

In addition, studies on the safety management of construction sites have also been conducted. The safety information that is generally used at construction sites does not reflect the factors related to the actual work environment; hence, it becomes more difficult to identify potential safety threats and provide the correct information to the right people at the right time [16]. Moreover, about 80% of all accidents occurring at construction sites are caused by dangerous actions of workers, and most deaths are caused by workers falling from high places or being hit by moving objects or construction equipment [17].

Methods for controlling and managing the behaviors of workers in the field, where it is becoming increasingly more difficult to secure the safety of the personnel, have been studied [18,19]. In contrast to such research trends, this study is aimed at promoting the rapid response to an impending collapse using an alarm or alert that does not interfere with the construction by developing a safety system that can be directly installed at a construction site [20,21].

In this study, focusing on the most dangerous accidents that occur at actual construction sites, i.e., collapses, a custom load-cell was developed using Bluetooth, along with a design to combine it with a prefabricated shoring system under rough conditions found at existing construction sites. This study is also aimed at investigating a system that can manage safety and prevent accidents through real-time Bluetooth load-monitoring using a product developed by Buildit, Inc. (Plaistow, NH, USA).

### Design Framework

**Figure 2:**
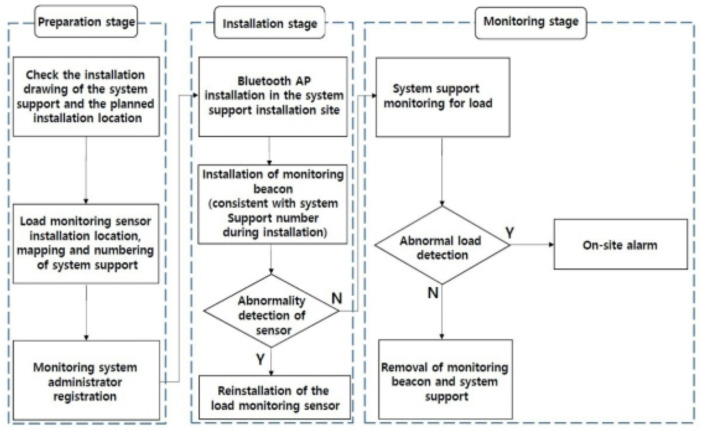
System support monitoring process.

Figure 2 shows the system-support monitoring process. During the preparatory step of step 1, you will review the system support installation drawing and the planned location of the wireless load-cell, and programmatically number the mapping and system support locations. Then, register as an administrator of the monitoring system and enter the site name and its section. The installation step in step 2 installs the onsite Bluetooth AP and surveillance beacon. Check the sensor installation abnormality at the site before placing, and if there is an abnormality, you will need to reinstall the load monitoring. In the monitoring step of step 3, system-support load-monitoring is performed when there is no abnormality in the sensor installation. When an abnormal load is detected, a warning sound is heard at the site, and when the color of the warning light changes from green to yellow to red, the worker interrupts the work and understands the cause. Due to the conservative approach of the gradual load value, even if the warning light turns red, it does not indicate the hypothetical structural collapse, and the operator is instructed to interrupt the work and disperse the load.

## 2. Methodology

### 2.1. Load-Cell Principle

A load cell is a transducer that measures physical quantities such as force or load by converting them into electric signals. Objects undergo deformation in proportion to the force or load applied to them, and the amount of deformation per unit length is called strain. Strain is characterized by a linear change in proportion to the force or load applied. Strain measurements are required based on engineering needs, and the sensor developed for such purpose is called a strain gauge. A strain gauge is based on the principle in which the electric resistance of an object depends on the change of its length and cross-sectional area. In other words, load cells convert a physical deformation occurring at the sensing element of the elastic strain member, which generates a structurally stable deformation allowing a force or load to change in terms of electric resistance, and then convert it into a precise electric signal through the configuration of an electric circuit called a Wheatstone bridge [22].

### 2.2. Strain Gauge-Based Load-Cell

A strain gauge uses the property of a metal or semiconductor that changes its resistance owing to a deformation that occurs when stress is applied. The resistance (*R*) of a metal conductor is expressed as follows:

(1) $$R=\rho \frac{L}{A}$$

where *ρ* is the specific resistance of the resistance wire and *A* and *L* are the cross-sectional area and length of the metal conductor, respectively.

For the gauge in a load cell, a wire gauge, a film gauge, or a foil gauge is used. A foil gauge is made of copper and nickel alloy. It is typically fabricated by processing a thin film with a thickness of 5–10 μm and attaching it with polyimide to a base using an adhesive followed by exposure and an etching process. The load cell is fabricated by processing an elastic strain member and attaching four strain gauges to a part where the largest strain occurs to measure the applied load based on a change in strain.

A change in the resistance of the strain gauge is directly related to the strain, according to the following equation:

(2) $$\frac{\Delta R}{R}=K\times \epsilon$$

where R is the resistance (Ω) before the occurrence of a strain, ΔR is the change in resistance (Ω) when a load is applied, K is a proportional integer (gauge rate of approximately 2), and *ϵ* is the strain (Δ*L*/*L*).

When a force is applied to the load cell, the resistance of the attached gauge is changed. This change in resistance is proportional to the applied force. Because the change in the resistance of the gauge is extremely small, the load cell is generally wired using the Wheatstone bridge circuit shown below.

**Figure 3:**
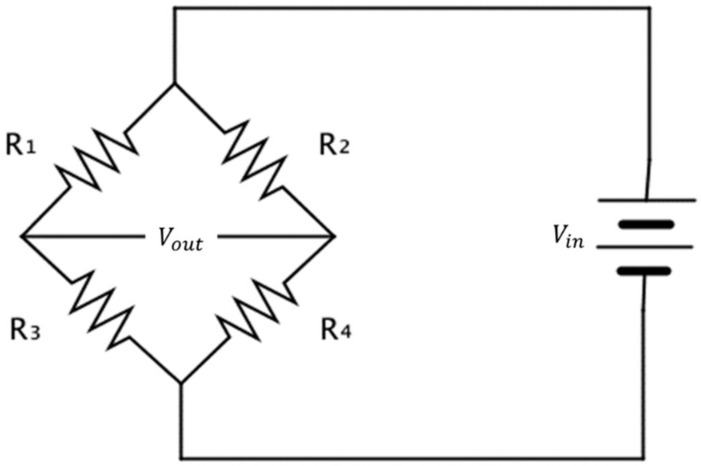
Wheatstone bridge circuit.

The Wheatstone bridge shown in Figure 3 is an electric circuit that detects slight changes in resistance. The change in resistance by strain is also converted using this circuit. The Wheatstone bridge is made up of four resistances. The gauges of $R_{1}$ through $R_{4}$ are attached as shown in the figure, and the output voltage before a load (force) is applied according to the electric flow can be expressed as follows:

(3) $$V_{out}=\frac{R_{1}R_{3}-R_{2}R_{4}}{\left(R_{1}+R_{2}\right)\left(R_{3+R_{4}}\right)}V_{in}$$

where $V_{out}$ is the load-cell output voltage and $V_{in}$ is the load-cell driving voltage.

When the resistance change occurs as $+\Delta R_{1}$, $-\Delta R_{2}$, $+\Delta R_{3}$, and $-\Delta R_{4}$ due to the strain applied to each gauge of the Wheatstone bridge ($R_{1}$ through $R_{4})$, the output voltage of the load cell is generated as the following expression:

(4) $$\Delta V_{out}=\left\{\frac{R_{1}R_{2}}{{\left(R_{1}+R_{2}\right)}^{2}}\left(\frac{\Delta R_{1}}{R_{1}}+\frac{\Delta R_{2}}{R_{2}}\right)\right\}V_{in}+\left\{\frac{R_{3}R_{4}}{{\left(R_{3}+R_{4}\right)}^{2}}\left(\frac{\Delta R_{3}}{R_{3}}+\frac{\Delta R_{4}}{R_{4}}\right)\right\}V_{in}$$

where if $R_{1}=R_{2}=R_{3}=R_{4}=R$, we obtain the following:

(5) $$\Delta V_{out}=\frac{\Delta R}{R}V_{in}$$

From (2), the following relationship is established: $\frac{\Delta R}{R}=K\times \epsilon$. Hence,

(6) $$\Delta V_{out}=K\times \epsilon \times V_{in}$$

Equation (6) shows that the output voltage ($V_{out}$) is proportional to the strain (ϵ) [23].

### 2.3. Design of Elastic Strain Member Using ANSYS Simulation Program

**Figure 4:**
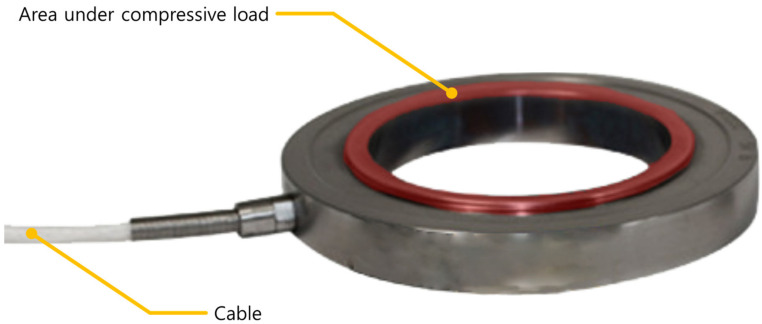
Commercial compression-type load-cells.

As can be seen from the above principle of a load cell, an elastic strain member that generates a structurally stable deformation in response to the applied force or load is required. Thus, to determine a load under the risk of collapse, we selected a compression-type load-cell shape as the basic frame as shown in Figure 4 for the elastic strain member receiving a compressive load.

**Figure 5:**
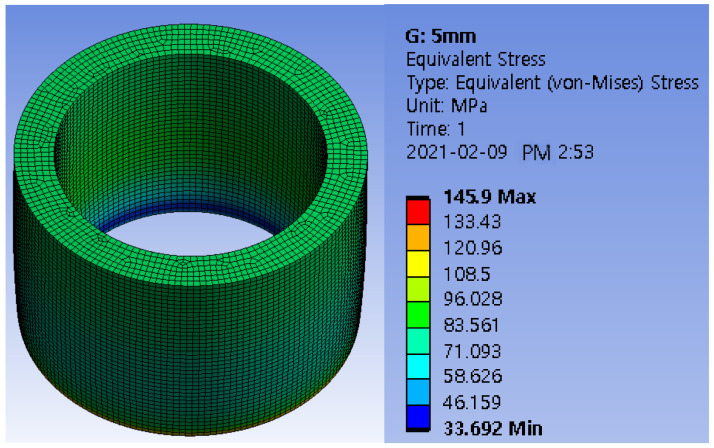
Finite element analysis of the ANSYS program.

As mentioned earlier, figures. In Figure 4, only the compressive load region was modeled to determine the safety factor of the elastically deformable member to prevent permanent deformation under a load of 5 tons. The inner diameter of the cylinder is fixed at 34 mm. Equation (7) determines the safety factor required for maximum stress and yield strength. The safety factor was determined by obtaining simulation values through finite element analysis using the ANSYS simulation program as shown in Figure 5 and Table 2. The safety factor can be determined according to the material and load condition [23], this study choose the safety factor as 1.5 due to the material is well-known steel and the load and stress can be easily determined. Therefore, a wall thickness of 5 mm was chosen.

(7) $$S=\frac{Yield\;Strength}{Max\;Stress}$$

Here, the yield strength is 250 MPa.

## 3. Hardware of the System

### 3.1. Load-Cell Shape

**Figure 6:**
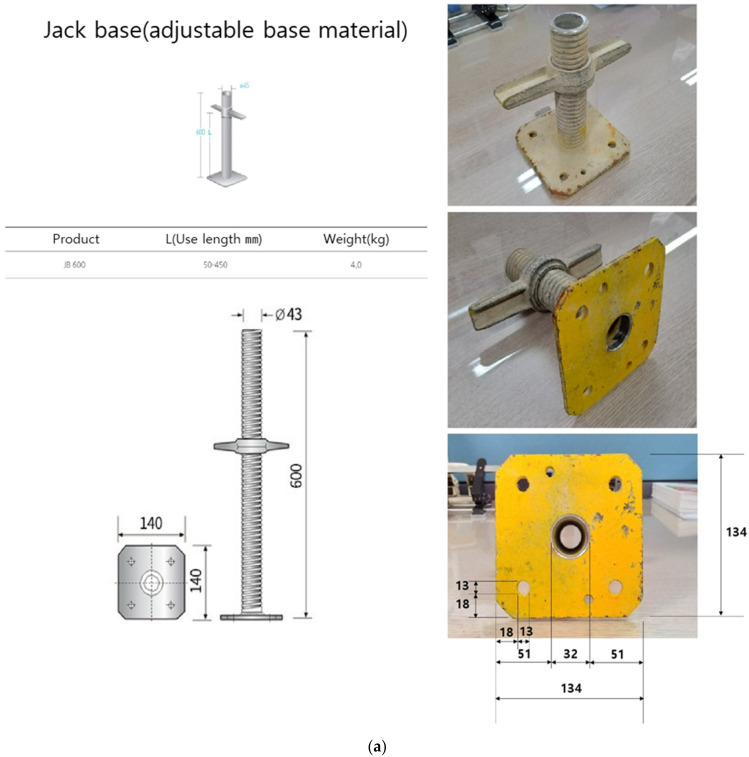

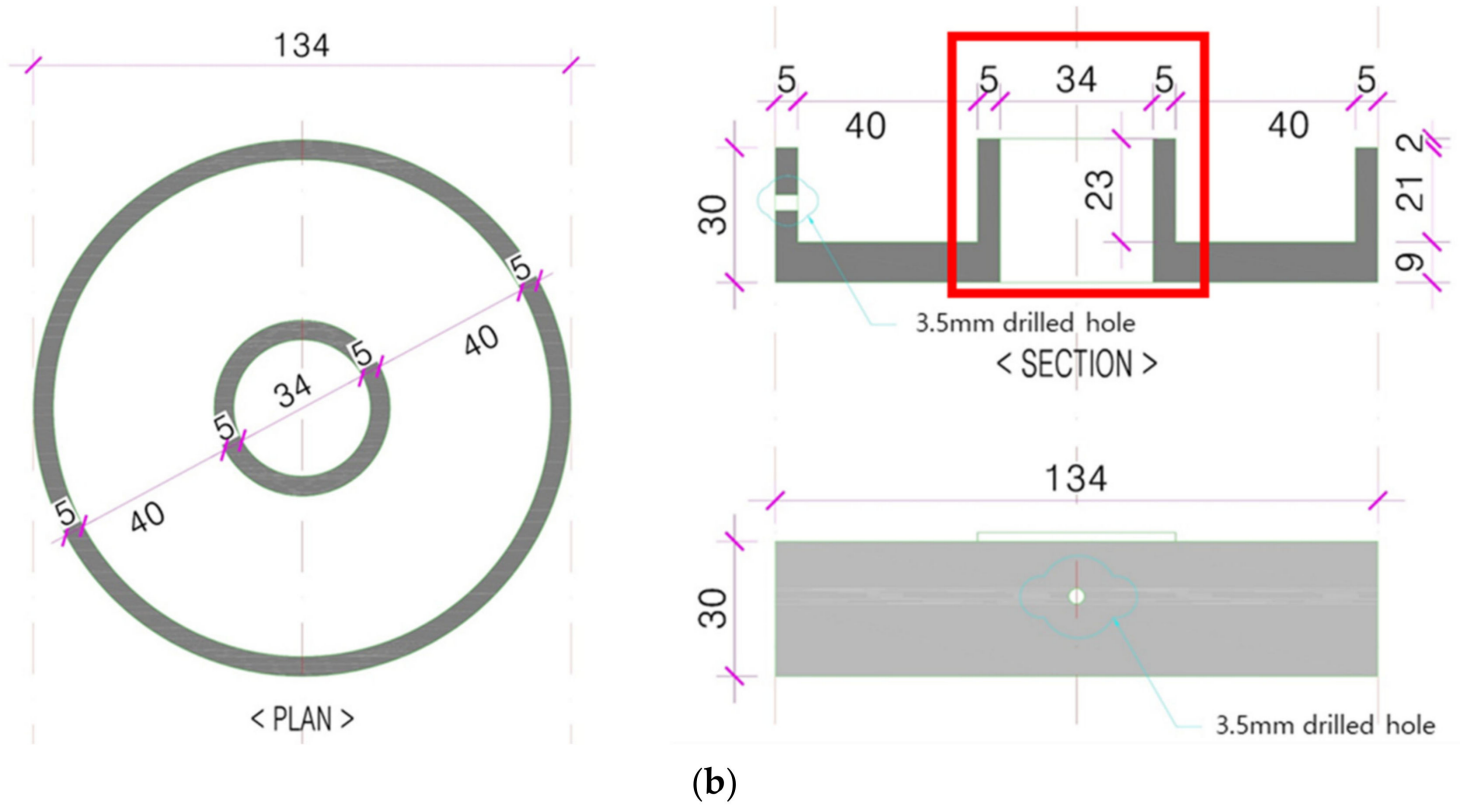
Scaffolding to which the prototype applies and drawings of the prototype. (**a**) Design of elastic strain member and Jack base: (bottom of system support/Jack base); (**b**) design of the elastic strain member.

**Figure 7:**
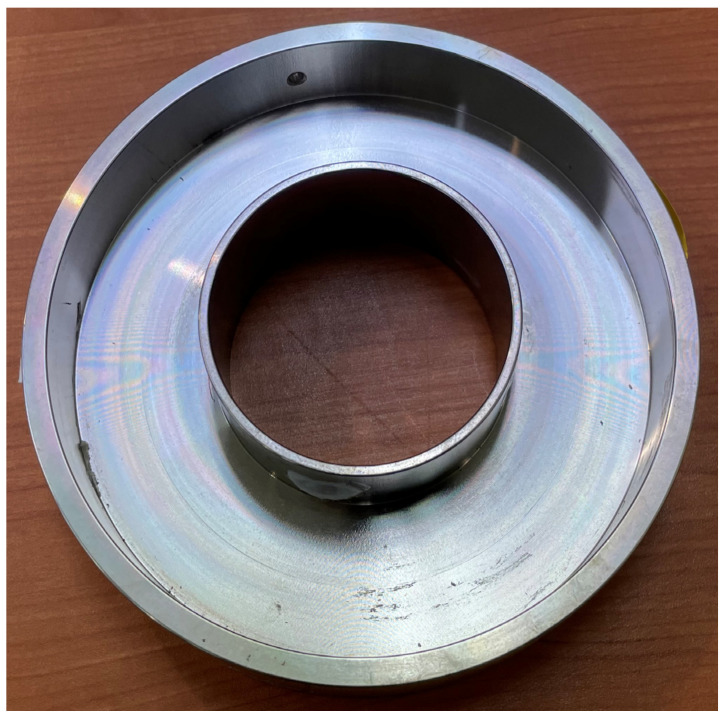
Prototype of elastic strain member.

The size of the elastic strain member and the space used to embed the Bluetooth module printed circuit board (PCB) were obtained by referring to Figure 6a. In addition, the part marked in Figure 6b (red box) was raised by 2 mm so that the load of the strain gauge would fully enter the center part. The elastic strain member was fabricated using SS400, which has a similar yield strength to the structural steel applied in the simulation, as shown in Figure 7.

**Figure 8:**
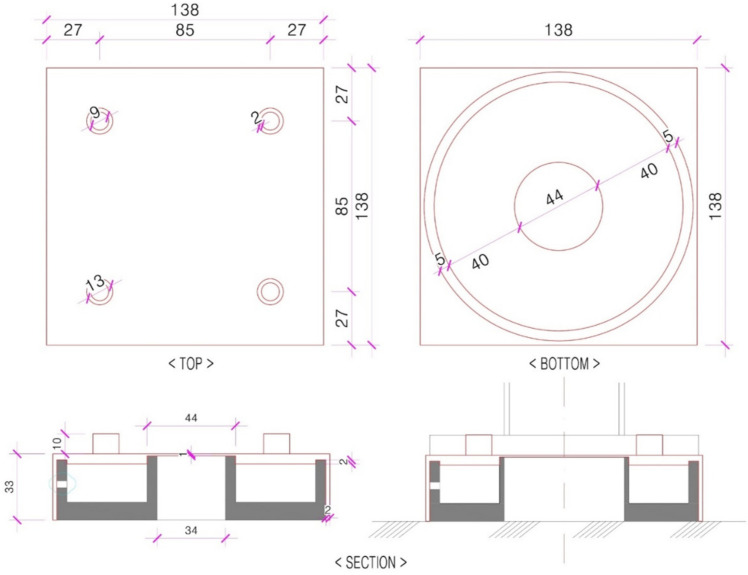
Load cell rubber casing design.

Figure 8 shows the rubber casing designed for waterproofing to prevent deformation by contaminants and external shock. It was designed to be fixed and prevent eccentricity by combining the protruding parts on the top of the rubber casing with the square holes of the Jack base. Furthermore, grooves were made on the inside of the casing for integration with the elastic strain member to prevent abnormalities in the load measurements.

### 3.2. Fabrication of Bluetooth-Based Wireless Load-Cell Prototype

**Figure 9:**
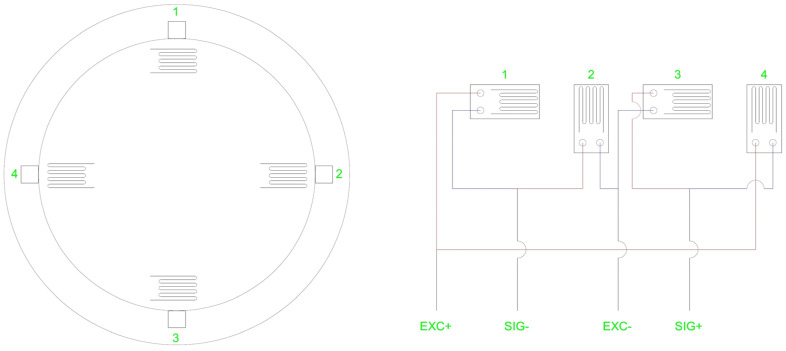
PCB Design and Circuit and Strain Gauge Attachment Directions(1: Horizontal, 2: Vertical, 3: Horizontal, 4: Vertical).

The Bluetooth-based wireless load-cell shown in Figure 9 is composed of an elastic strain member, a strain gauge, and a PCB. The changes in the load to the load cell make the strain changes in the elastic strain member and the strain gauge, which is attached to the elastic member, measure the change of strain.

In Figure 9, EXC± is a temperature compensation dummy, the voltage difference of which is sensed using SIG±. Because the change in voltage difference by weight is extremely small (approximately 0.007 V), the voltage difference of SIG± is amplified to 600 times the existing value using an AMP and the voltage is delivered to the ADC (14-bit) of the SoC (System on Chip).

**Figure 10:**
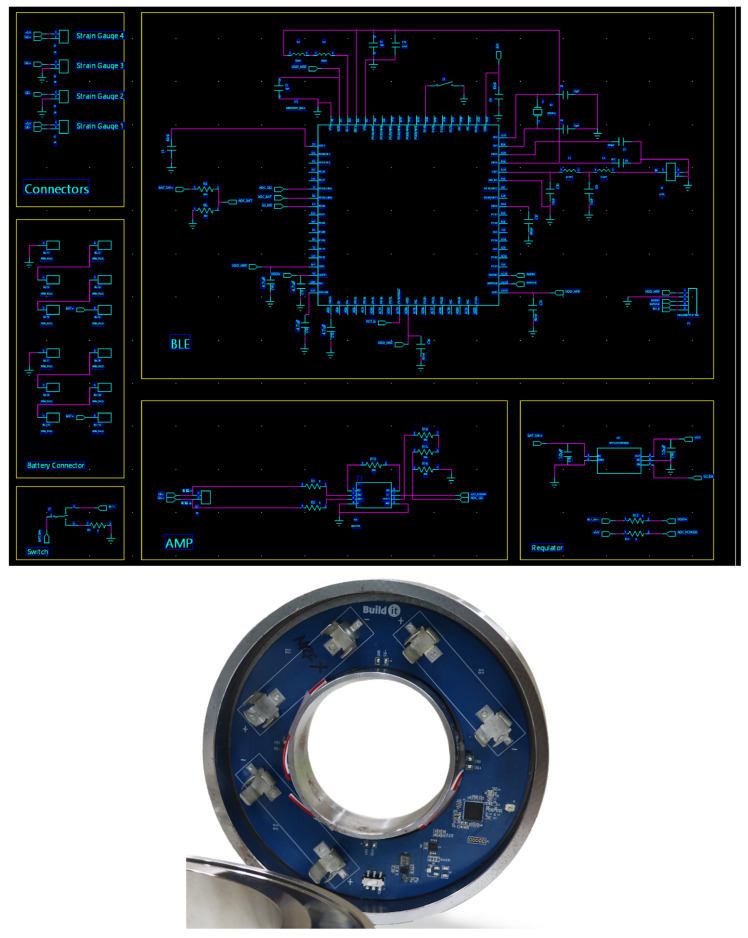
PCB design and circuit diagram.

As shown in the circuit diagram in Figure 10, the sensing value is delivered from the pressure sensor to the nRF52833 SoC embedded with a Bluetooth function. These sensing data are transmitted through the circuit using a Bluetooth Advertising Packet.

A Bluetooth Advertising Packet has an advantage in that communication at a similar level can be achieved at a much lower power than with a conventional method using the Bluetooth Low Energy (BLE) protocol from Bluetooth 4.0 released in 2010. We selected an Advertising Packet because it can transmit data without Bluetooth pairing and can prolong the battery life.

If you check the full data sheet of NRF52833, we provide 14-bit adc. In addition, the amp that provides 600 times gain is Texas Instruments’ INA333, which is a device that provides Low Quiescent Current: 50 μA, up to 1000 times gain. As an energy breakdown, 10 ms (about 15.9 mA) is used every 200 ms through strain-gauge measurement, NRF52833 ADC, 5 V burst regulating, and AMP power supply. BLE every 200 ms, data advertising (80 μA).

Timer, Interrupt and Pin Control 20 μA, (16 × 0.005) + (0.1 × 0.195) × 5 = consumes approximately 500 μAh AA batteries in parallel connection to approximate””ly 4800 mAh (6000 mAh, but excluding 20% due to discharge rate) 500 μAh × 24 × 365 = 4380 mAh, usable for 1 year.

When extending the battery life, it is possible to extend the test by lowering the measurement voltage of the strain gage being measured to 5 V with the step-up regulator and lowering the measurement time of 5 ms further.

Since the battery is non-replaceable, the battery can last longer without replacement after system installation. When applied, it is theoretically possible to operate with 3 AA batteries, and it is possible to measure once every 2 s and without replacing the batteries for more than a year by setting the communication cycle.

**Figure 11:**
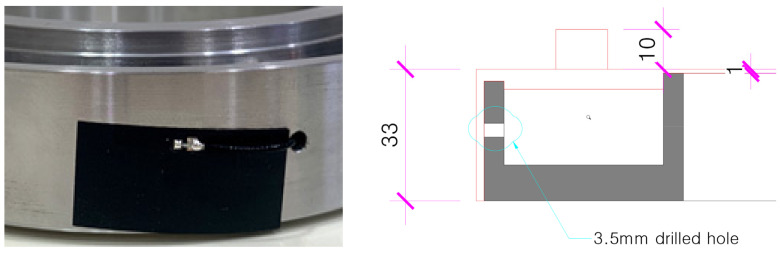
Installation of film antenna.

**Figure 12:**
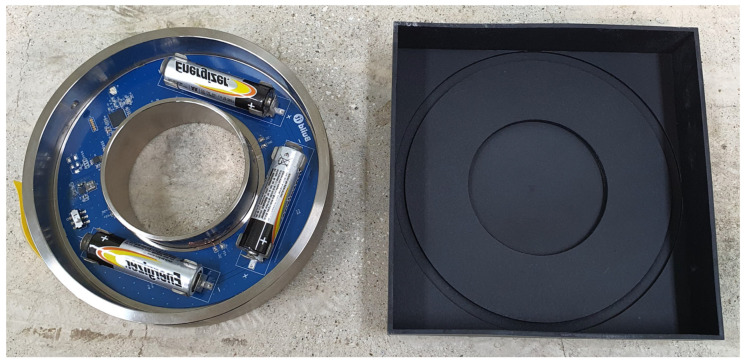
Wireless Bluetooth load-cell prototype and rubber casing.

Afterwards, it was found that the transmission range of the antenna for transmitting the internal strain gauge value was short due to the outer wall of the elastically deformable member. In order to prevent the load reception interference of the elastically deformable member, as shown in Figure 11, a drill hole was drilled in the outer wall to install the film antenna, and the waterproof finish was treated with silicone to complete the prototype of the wireless Bluetooth load-cell, as shown in Figure 12.

### 3.3. Development of IoT AP and Prototype Achieving Bluetooth Signal Reception and LTE Communication

The gateway, which is responsible for receiving Bluetooth signals and conducting LTE communication, refers to the software enabling communication between different types of networks by appropriately converting the different communication protocols.

**Figure 13:**
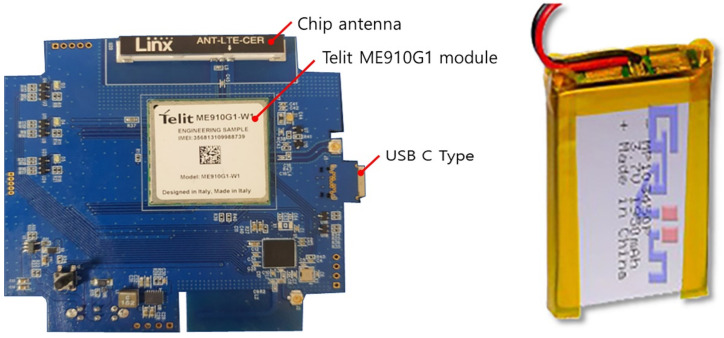
Gateway PCB and lithium battery.

We apply a wireless BLE TO LTE gateway, which converts the BLE protocol of Bluetooth into MQTT, HTTP, or HTTPS using the parts shown in Figure 13, along with an LTE USIM instead of Wi-Fi and Ethernet.

This makes it possible to supply a higher voltage and battery capacity than a basic 3.7 V AA battery while minimizing the gateway shape. In addition, the portability and communication aspect of the gateway is improved by using the charging function using a lithium-ion battery.

Lithium-ion polymer batteries are lithium-ion secondary batteries that use a polymer-based electrolyte. The explosive electrolyte of a lithium-ion battery has been changed to a solid electrolyte (polymer). Although the ionic conductivity, temperature characteristics, and lifespan are lower than those of liquid electrolytes, they have advantages such as safety, miniaturization, and the possibility of producing desired shapes. Additionally, these batteries can be produced in curved or thin forms for application in mobile devices such as smartphones and laptops. Therefore, we have chosen a lithium-ion polymer battery with high compatibility.

**Figure 14:**
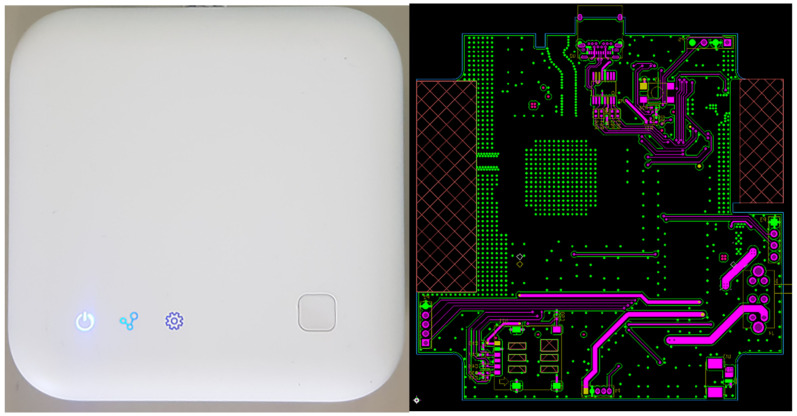
IoT AP prototype with the ability of LTE communication.

As shown in Figure 14, the condition of the gateway can be checked using three LEDs. The blue LED illuminates during normal operation, and the red LED illuminates when the power supply is defective or when a dangerous error occurs. In addition, the blue LED blinks repeatedly during normal data communication and the red LED illuminates when the data transmission has an error. The red LED also illuminates when the connection to the config prefabricated shoring system monitoring cloud is abnormal, the LTE communication signal is weak, or a USIM recognition error occurs.

Furthermore, a nano USIM was used, which was registered with a telecommunications company in advance after mold injection and outer-shape fabrication. After the USIM was recognized, the communication strength and registration status were checked through the base station, and the status was displayed through the LED indicators. The outer shape was produced by injection through molding. The LED indicator part was laser cut after being painted gray for visibility, and the case was painted white.

**Figure 15:**
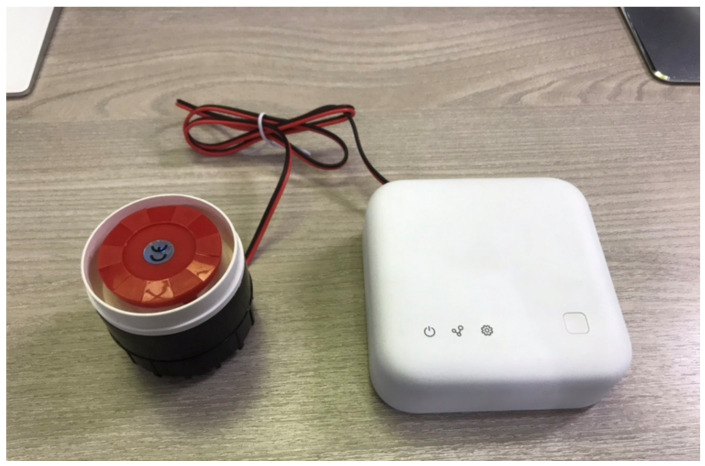
Gateway connected with an alert buzzer.

In addition, a method for instantly notifying the on-site workers using a buzzer installed at the site was necessary. Thus, as shown in Figure 15, a buzzer was connected to the gateway and the firmware was set to sound the buzzer in the event of danger.

### 3.4. Experiment on Prototype Performance Using UTM

**Figure 16:**
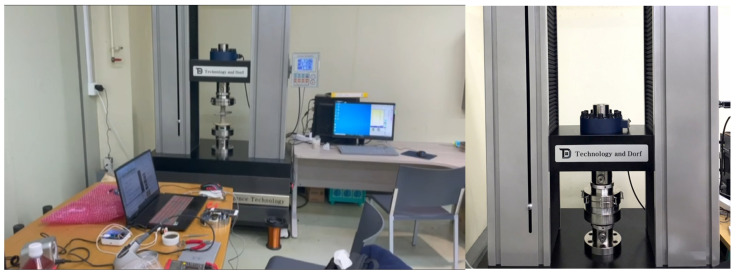
Load-cell compressive load experiment using UTM.

In order to verify the performance of the prototype and to calibrate a number of additional prototypes, compression experiments were performed using a universal testing machine (UTM) as shown in Figure 16. This experiment compares the value of the output voltage when the load is increased. In this experiment, loads from 0.2 tons to 5 tons were individually applied based on the load of the prefabricated support system.

As shown in Table 3, the initial output voltage varied by device owing to the different overall initial values, and the increased value also varied even when the same compression load was applied.

**Figure 17:**
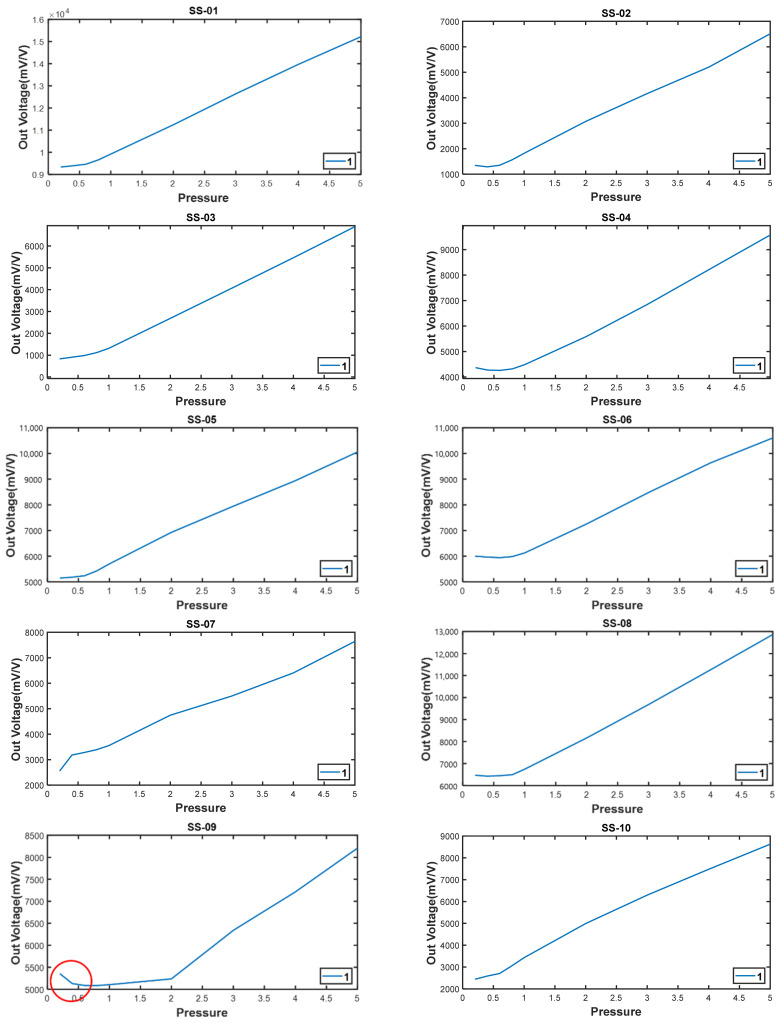

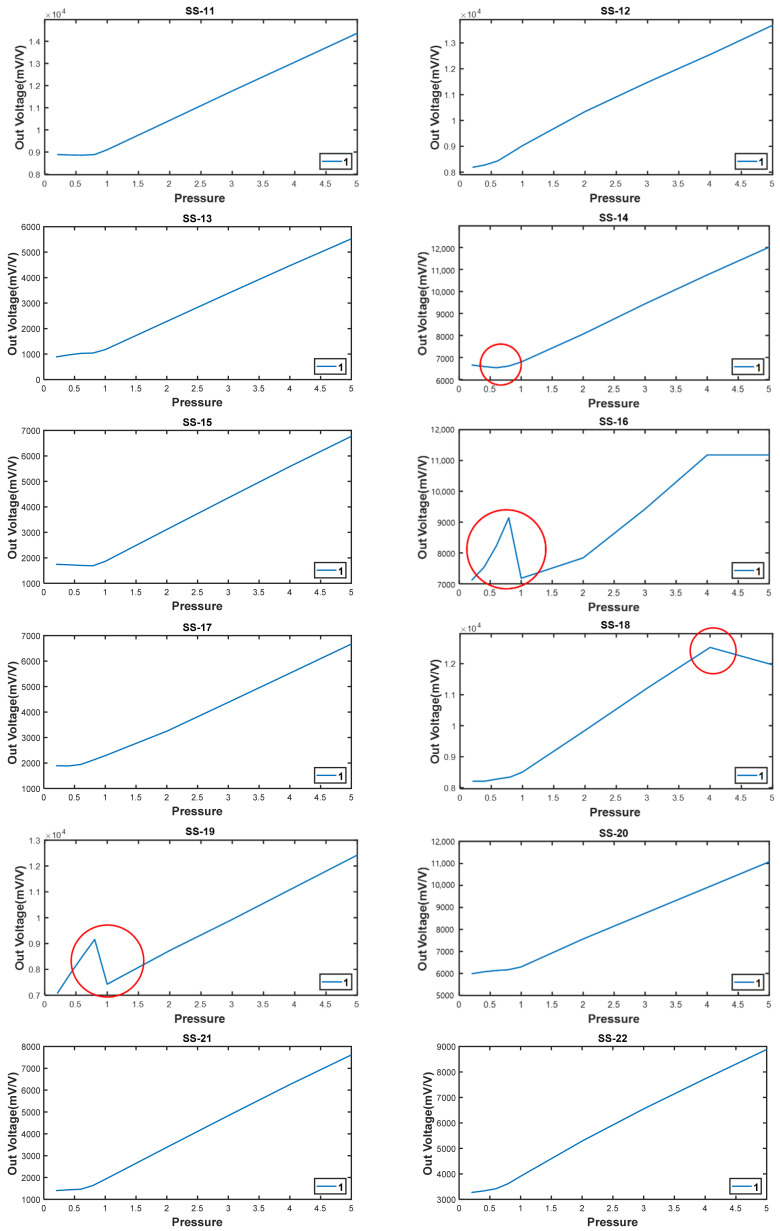

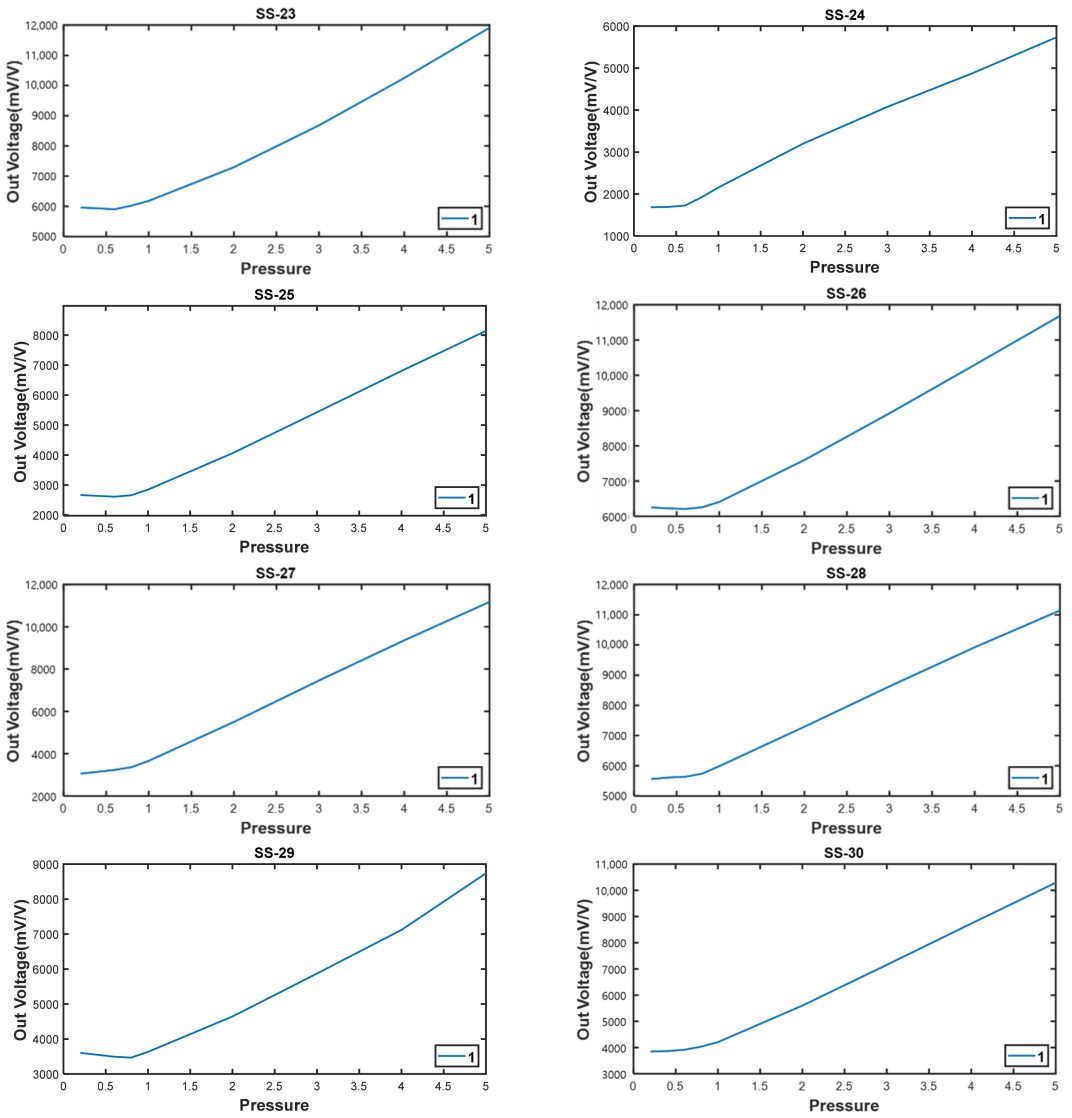
Graph of the data of the compressive load-cell experiment using UTM.

The data of some parts marked by the red circles in Figure 17 were relatively non-uniform under light loads until 1 ton was applied; however, after 1 ton, the load increased linearly as the load entered the dangerous zone. This indicates a strong influence of buckling owing to the small thickness of the inner diameter.

As a result of this influence, an average accuracy of 0.25% was obtained.

Although there has minor errors over that range (0 to 1 and over 4 tonf), but it can be ignored because the load which acts to the system support is up to 1 tonf and under 4 tonf. So, an additional action was taken because it could be adjusted through firmware calibration.

### 3.5. Remote Load-Monitoring Program for a Prefabricated Shoring System

The personal server was opened by entering the assigned ID, and the locations where the sensors were installed were marked on the drawing for efficient monitoring. In this manner, the system was developed to enable the overall monitoring on the screen and the storage of massive data that are recorded every second.

**Figure 18:**
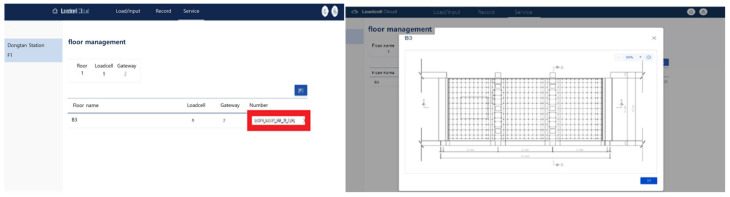
Uploading of the construction drawings.

**Figure 19:**
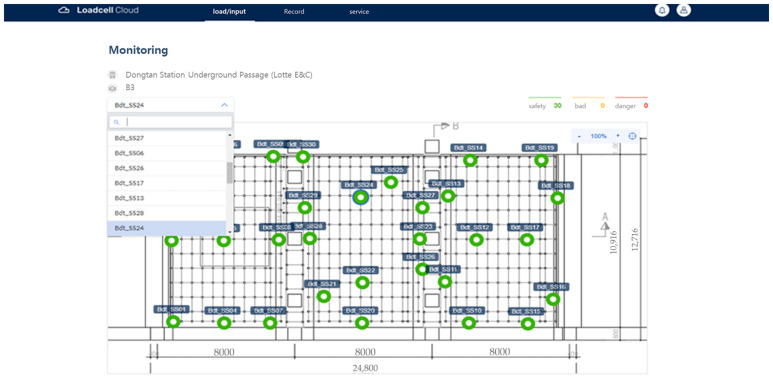
Setting the locations of devices using drag and drop.

Through the prefabricated shoring system load-monitoring program, the designated administrator can collect the data of the Bluetooth wireless load-cell received at the AP. The drawings are uploaded as shown in Figure 18, and the data of each load cell can be displayed in real time on the program by setting the installed locations through a drag and drop, as shown in Figure 19.

The current status, including safety, warning, and danger alerts, can be displayed using the range set as the maximum load of the prefabricated shoring system with the data obtained from structural calculations at the time of the site design. The safety load (1–2 tons) was set to be displayed in green, the warning load (2–3 ton) in orange, and the dangerous load (3–5 tons) in red.

After completing the installation and settings, the required data—including real-time load data, abnormal load alert pushes, and abnormal load alert history—can be displayed on the program.

**Figure 20:**
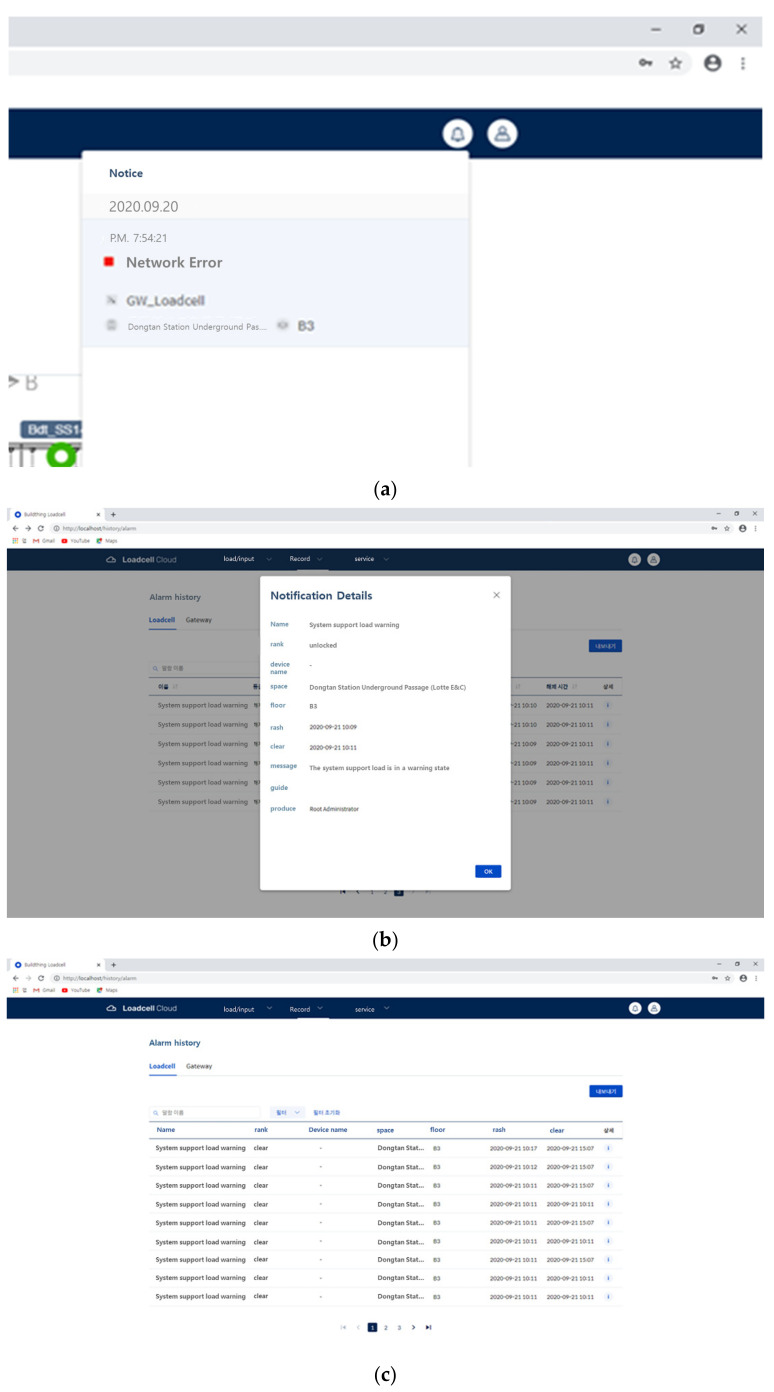
Monitoring screens (**a**) Notification push; (**b**) categorization of notification history; (**c**) viewing notification history.

As shown in Figure 20, when an abnormal situation occurs, the logs are recorded. Because the app push messages of administrators and the logs of the program are stored, the cause can be analyzed in the event of an accident, and immediate responses to dangerous situations are made possible.

## 4. Final Validation through On-Site Testing

### 4.1. Modeling Analysis of System Support

**Figure 21:**
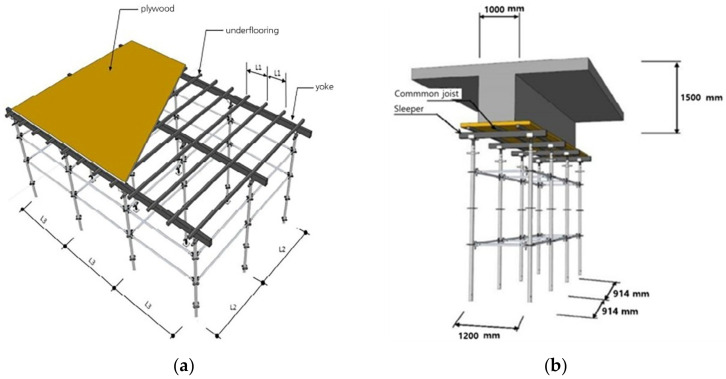
Slab, beam pouring section detail view. (**a**) Detailed view of slab-pouring section; (**b**) detailed view of beam-pouring section.

**Figure 22:**
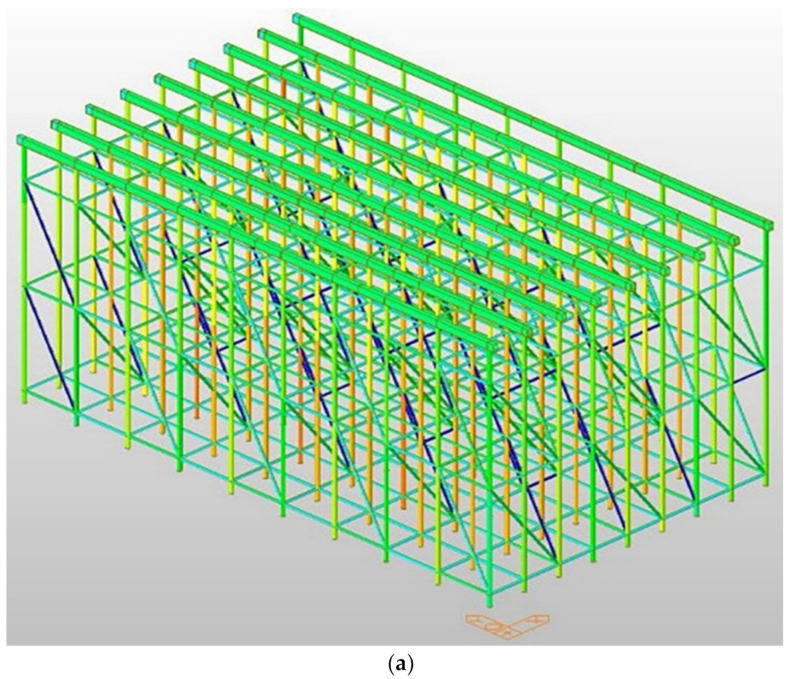

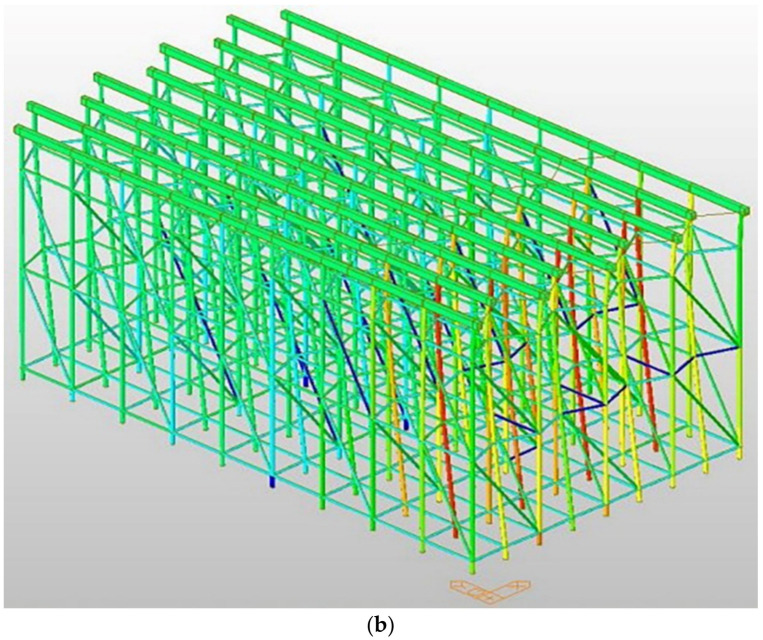
Distributed pour and concentrated pour 3D modeling review. (**a**) Distributed pour-stress state; (**b**) pour-stress state.

Figure 21 details the beam section’s joists, yokes, and system support. In order to secure structural safety when pouring concrete, it is appropriate to calculate the joist, yoke, and system-support interval according to the beam size (1000 × 1500) so that the site safety manager can check the installation interval between the joists, yoke, and system support. As shown in Figure 22, modeling review was conducted for concrete-distributed pouring and concentrated pouring. As shown in Figure 22a, in the case of distributed pouring, concentrated stress does not occur in the system support. As shown in Figure 22b, it was confirmed that the vertical load rises rapidly during concrete pouring, and concentrated stress is generated in the system support, which can lead to the collapse of the temporary facility.

Figure 21 details the beam section’s joists, yokes and system support. In order to se-cure structural safety when pouring concrete, it is appropriate to calculate the joist, yoke, and system support interval according to the beam size (1000 × 1500) so that the site safety manager can check the installation interval between the joists. yoke and system support. As shown in Figure 22, modeling review was conducted for concrete distributed pouring and concentrated pouring. As shown in Figure 22a, in the case of distributed pouring, concentrated stress does not occur in the system support, As shown in Figure 22b, it was confirmed that the vertical load rises rapidly during concrete pouring, and concentrated stress is generated in the system support, which can lead to the collapse of the temporary facility.

### 4.2. Test Results and Discussion

**Figure 23:**
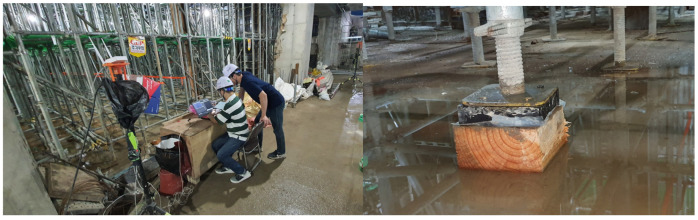
Field application of the Bluetooth load measurement monitoring system.

The field verification of the wireless load measurement monitoring system was applied and evaluated in the field where the system support was used, as shown in Figure 23. In order to observe the load change, concrete was poured on the upper part of the system support and the load change pattern by position was investigated in the installation section. The casting time took about 3 h and 30 min in total.

**Figure 24:**
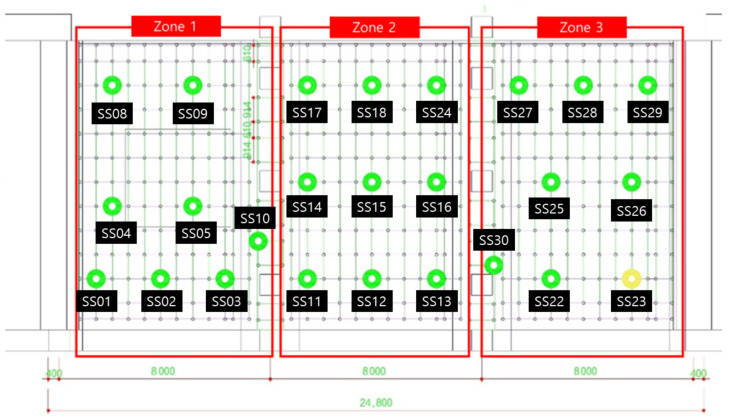
Load-monitoring screen (11:30).

Since LTE reception was not possible at the underground site, we connected the AP in series with the laptop computer so that the gateway could monitor the signals sent to and received from the Bluetooth beacon. Figure 24 shows the real-time load-monitoring screen.

**Figure 25:**
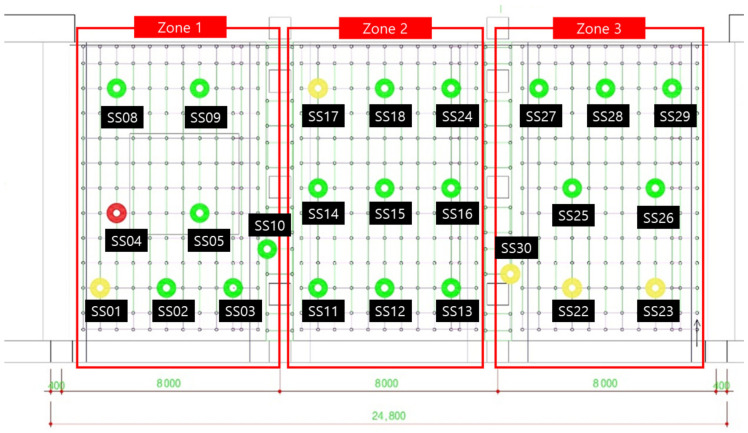
Load-monitoring screen (15:00).

Figure 25 shows the color change of each device according to the load change as the concrete placement progresses. In each area, after pouring concrete, 3–4 workers will work to fill every corner with a vibrator. During this process, it was confirmed that at the beginning of the first injection, a red mark (dangerous load) appeared on one load cell when workers leaned against and piled up concrete high. However, it was confirmed that the concrete turned yellowish green because the concrete spread evenly around it. (The range of the color stage is set a little conservatively, so just because the red mark is displayed does not mean that it will collapse immediately.) 

**Figure 26:**
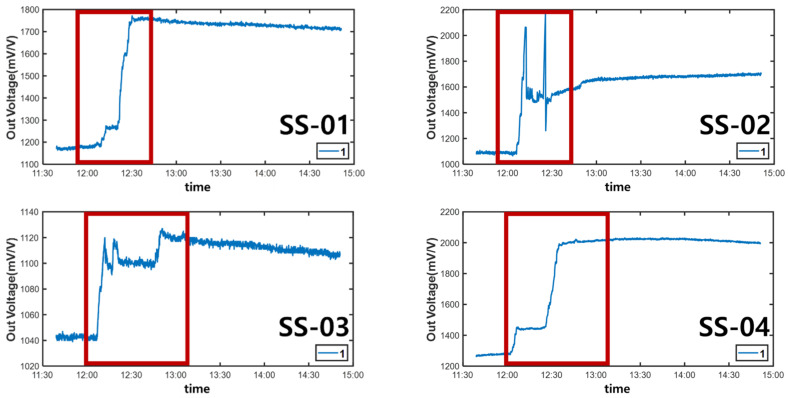

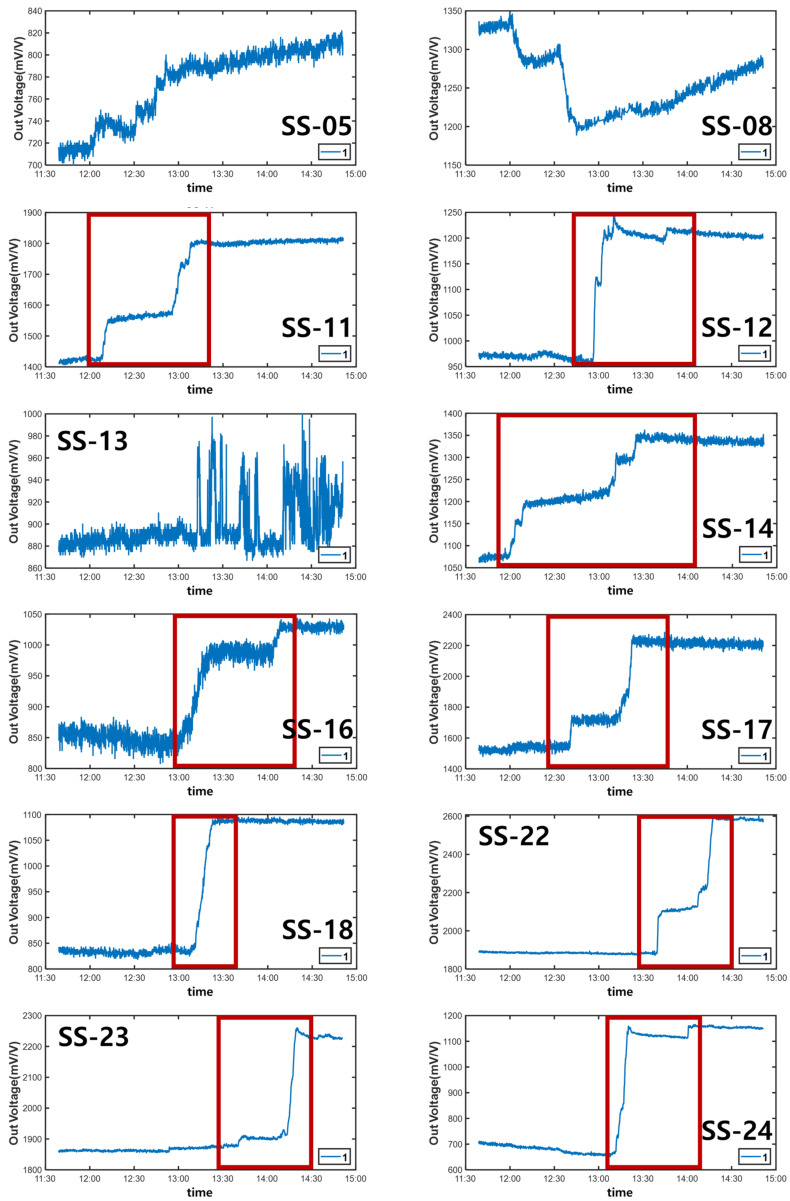

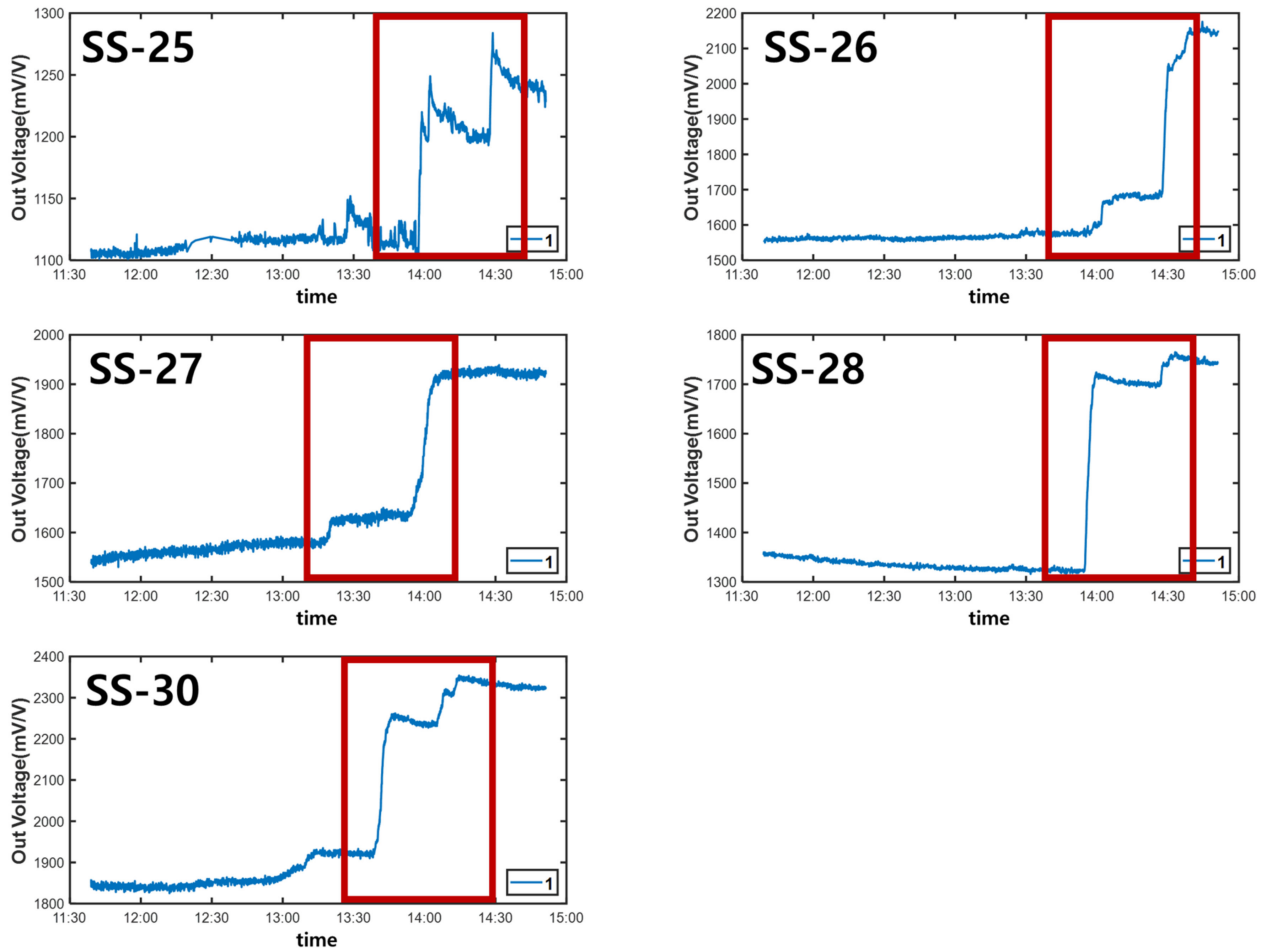
Monitoring data graphs for 11:30–15:00.

After the experiment, a large amount of data transmitted from the Bluetooth beacon was graphed using MATLAB as shown in Table 4. As shown in Figure 26, the values on the *X*-axis represent time and the values on the *Y*-axis represent the output voltage. As shown in the figure, the amount of change over time in the graph was confirmed by dividing the left (morning) part and the right (afternoon).

In Figure 26, the red box shows the significantly increased range. It can be seen that the output voltage value increases in the order of zone 1, zone 2, and zone 3 according to the injection direction. However, in the case of SS-05, SS-08, and SS-13 in Figure 26, the graph was not displayed properly due to buckling and eccentricity.

Through these field experiments, it was confirmed that when the poured concrete was flattened with a vibrator and a large amount was poured into every corner, the load-cell load value at that location changed and the load value increased or decreased.

**Figure 27:**
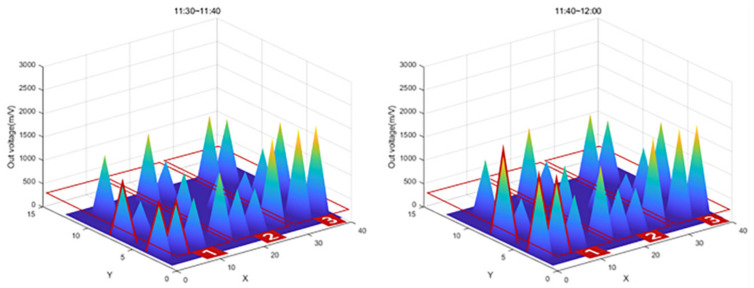
Sum of 3D graph values for data from 11:30–12:00.

**Figure 28:**
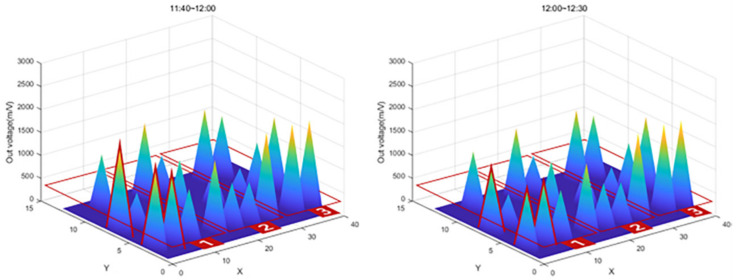
Sum of 3D graph values for data from 11:40–13:30.

**Figure 29:**
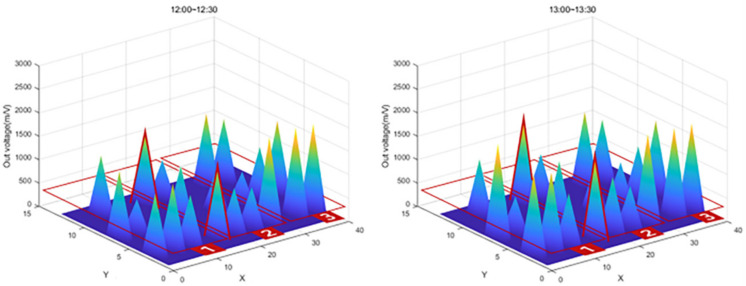
Sum of 3D graph values for data from 12:00–13:30.

**Figure 30:**
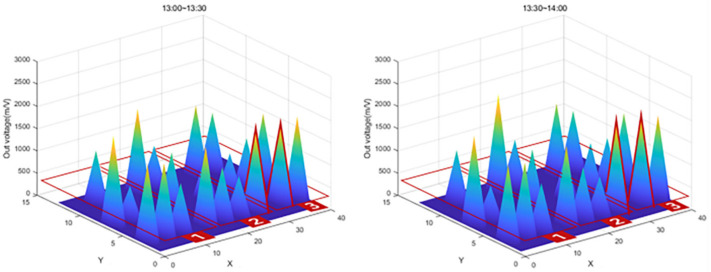
Sum of 3D graph values for data from 13:00–14:00.

**Figure 31:**
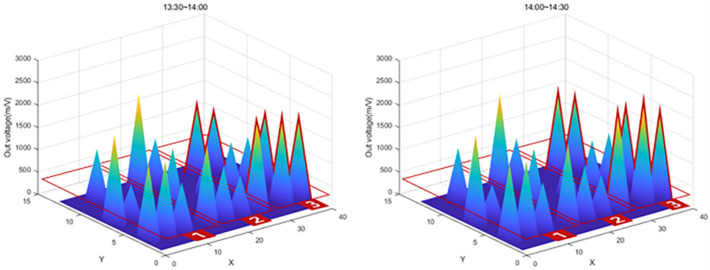
Sum of 3D graph values for data from 13:30~14:30.

**Figure 32:**
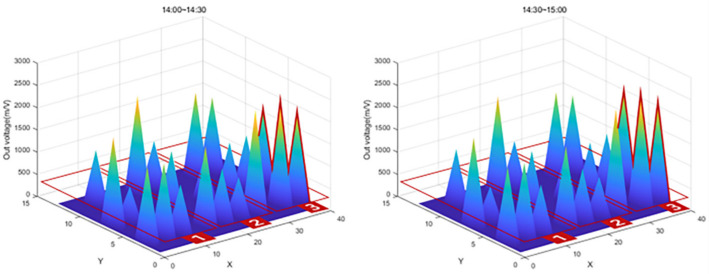
Sum of 3D graph values for data from 14:00~15:00.

The graph in Figure 27 shows that the value of Zone 1, the first concrete injection zone, has increased significantly. Referring to Figure 28, it can be seen that the load is slightly reduced by load balancing by using the vibrator for leveling work with a tightly packed concrete. In the graph in Figure 29, you can see that the load gradually increases as the concrete placement work moves to Zone 2. Figure 30 also shows that the load on the top slab of the column affects the load cells placed around it by casting. The columns between the zones, and the load, will increase slightly compared to the surroundings. The graphs in Figure 31 and Figure 32 show that the load gradually increases as the concrete injection operation moves from Zone 2 to Zone 3.

## 5. Conclusions

In this study, a Bluetooth load-cell-based safety management support-monitoring system was established. Both hardware and software approaches have been implemented. The remote load-monitoring program intuitively shows the level of danger, and when a dangerous load is detected at the construction site, it immediately sounds a buzzer to help with emergency evacuation. It is meaningful in securing the safety of workers by preventing the repeated and minor collapse accidents of foundation construction. The main tasks of this study are as follows:

1. Developed wirelessly, so that it does not interfere with the construction site, and a custom Bluetooth load-cell has been developed.
2. A load was applied to the prototype with UTM, and an experiment was performed to confirm the operation and to calibrate.
3. In the first UTM experiment, the accuracy was very low with an average value of 0.25%, so to correct this, the firmware of each prototype was modified and the experiment was conducted again to develop the finished product.
4. We developed an AP that can transmit and receive data from the load cell and software that can intuitively view data. The user can control the range of safety, warning, and danger in the system setting and set the position of the product in the form of Drag&Drop according to the drawing.
5. A field experiment with the completed Bluetooth load-cell was conducted in Dongtan, Korea, and the overall data showed validity according to the applied load. However, there was an eccentricity between the load cell and the prefabricated shoring system due to the incorrect location because of the poor conditions of the temporary facility and radio-wave interference caused by temporary iron poles at the site. Moreover, the load-bearing part of the elastic body was thin and prone to buckling. These led to a slight error in the data.

Through this, it has been proven that the monitoring system can be used in the construction field. We will proceed with improving the accuracy by modifying the design of the elastic body so it can be applied to other types of supports in our future research. We hope that it contributes to the opportunity for a safety inspection to realize real-time monitoring by using wireless sensor networks for the Internet of Things era.

## Figures and Tables

**Table 1:** Major accidents by year.

	Manufacturing	Construction Industry	Service Industry	ETC
**Total**	117	188	39	17
**2009**	11	15	2	2
**2010**	10	17	5	2
**2011**	12	20	9	0
**2012**	21	30	6	1
**2013**	16	19	2	3
**2014**	11	13	2	2
**2015**	7	16	3	2
**2016**	14	17	3	2
**2017**	7	21	3	3
**2018**	8	20	4	0

**Table 2:** Result of safety factor calculation using ANSYS program.

Wall Thickness (mm)	Strain (mm/mm)	Stress (MPa)	Safety Factor
2	1.7572 × 10^−3^	351.39	0.71
3	1.1205 × 10^−3^	224.10	1.12
4	8.7396 × 10^−4^	174.79	1.43
**5**	**7.2949 × 10^−4^**	**145.90**	**1.71**
6	5.9500 × 10^−4^	119.00	2.10

**Table 3:** Data from compressive load experiment using UTM.

Load Cell	0.2 T	0.4 T	0.6 T	0.8 T	1 T	2 T	3 T	4 T	5 T
SS-01	9335	9393	9461	9656	9921	11,242	12,642	13,971	15,219
SS-02	1344	1291	1350	1559	1822	3071	4163	5201	6513
SS-03	833	908	982	1117	1312	2687	4071	5460	6887
SS-04	4370	4273	4257	4320	4487	5587	6857	8218	9583
SS-05	5146	5180	5236	5430	5700	6927	7947	8942	10,055
SS-06	6000	5962	5942	5983	6126	7256	8483	9637	10,604
SS-07	2556	3181	3280	3390	3554	4745	5500	6407	7641
SS-08	6469	6425	6449	6493	6745	8165	9687	11,266	12,863
SS-09	5356	5130	5087	5085	5105	5236	6341	7214	8206
SS-10	2439	2587	2703	3055	3433	4992	6293	7476	8628
SS-11	8889	8867	8859	8887	9104	10,428	11,759	13,058	14,364
SS-12	8187	8280	8433	8725	9027	10,344	11,478	12,552	13,684
SS-13	888	967	1030	1040	1178	2282	3383	4468	5525
SS-14	6658	6588	6533	6610	6795	8065	9442	10,750	12,013
SS-15	1747	1728	1704	1688	1864	3116	4357	5584	6770
SS-16	7112	7534	8232	9143	7177	7840	9430	11,179	0
SS-17	1893	1886	1943	2112	2292	3251	4384	5523	6670
SS-18	8212	8212	8284	8343	8507	9850	11,218	12,525	11,963
SS-19	7065	7800	8495	9159	7428	8731	9934	11,180	12,424
SS-20	5987	6076	6133	6172	6298	7568	8736	9903	11,073
SS-21	1403	1440	1461	1635	1919	3384	4832	6254	7611
SS-22	3269	3331	3419	3620	3907	5288	6550	7732	8881
SS-23	5959	5934	5904	6025	6181	7295	8683	10,248	11,911
SS-24	1683	1692	1723	1919	2158	3200	4078	4871	5731
SS-25	2670	2636	2612	2659	2847	4060	5430	6808	8140
SS-26	6250	6225	6270	6255	6407	7596	8924	10,297	11,686
SS-27	3058	3142	3234	3364	3663	5502	7464	9363	11,176
SS-28	5557	5611	5636	5738	5896	7292	8631	9923	11,140
SS-29	3603	3548	3495	3466	3631	4645	5873	7120	8741
SS-30	3849	3864	3915	4033	4207	5601	7156	8731	10,289

(SS: System Support/unit: outvoltage).

**Table 4:** Data on remote load-monitoring measurements.

Load Cell	11:40	12:00	12:30	13:00	13:30	14:00	14:30	15:00
SS-01	1173	1174	1327	1754	1738	1733	1724	1715
SS-02	1089	1090	1429	1600	1665	1679	1686	1696
SS-03	1042	1043	1087	1108	1117	1115	1111	1108
SS-04	1267	1274	1434	1977	2021	2024	2017	2003
SS-05	709	714	733	764	789	796	803	808
SS-08	1326	1330	1295	1232	1218	1229	1256	1275
SS-11	1416	1422	1508	1577	1783	1801	1807	1810
SS-12	971	971	972	981	1206	1206	1208	1204
SS-14	1067	1075	1178	1208	1280	1344	1337	1335
SS-16	855	857	851	841	934	989	1021	1029
SS-17	1529	1520	1541	1655	1893	2221	2214	2206
SS-18	837	834	832	834	954	1088	1087	1086
SS-22	1892	1888	1885	1884	1880	2029	2379	2583
SS-23	1860	1863	1862	1863	1872	1894	2060	2230
SS-24	705	700	687	666	884	1116	1156	1153
SS-25	1105	1106	1108	1117	1121	1125	1213	1244
SS-26	1555	1560	1563	1561	1567	1578	1688	2118
SS-27	1542	1550	1561	1574	1596	1643	1913	1922
SS-28	1357	1352	1341	1331	1327	1375	1707	1749
SS-30	1846	1842	1842	1855	1908	2121	2309	2327

(unit: outvoltage).
